# Influence of the artificial turf certification on physical performance and muscle damage in football players (QUALTURF PROJECT)

**DOI:** 10.1038/s41598-021-88192-w

**Published:** 2021-04-21

**Authors:** Javier Sanchez-Sanchez, Jose Luis Felipe, Antonio Hernandez-Martin, David Viejo-Romero, Vicente Javier Clemente-Suarez, Leonor Gallardo, Jorge Garcia-Unanue

**Affiliations:** 1 Faculty of Sport Sciences, Universidad Europea de Madrid, Madrid, Spain; 2 Grupo IGOID, University of Castilla-La Mancha, Toledo, Spain

**Keywords:** Predictive markers, Physiology, Materials science

## Abstract

This study aimed to analyse the influence of the FIFA Quality PRO certification of artificial turf pitches on the physical, physiological performance and muscle damage in soccer players. Fifteen healthy male players (21.2 ± 1.4 years; 178.2 ± 4.3 cm; 79.1 ± 8.3 kg) from a university football team were selected to participate in the research. Mechanical properties related to surface–player interaction were assessed on the two surfaces selected for this study. A randomized design was used and the players performed the Ball-sport Endurance and Sprint Test (BEAST90) on the different artificial turf fields. Average time of the 20 m sprints was longer on the FIFA Quality Pro surface than on the non-certified pitch (+ 0.13 s; *p* < 0.05; CI 95% − 0.01 to 0.27; ES: 0.305). The players’ perceived effort was higher in the first (+ 2.64; *p* < 0.05; CI 95% 0.92 to 4.35; ES: 1.421) and the second half (+ 1.35; *p* < 0.05; CI 95% − 0.02 to 2.72; ES: 0.637) of the test on the FIFA Quality Pro field. Comparative analysis between surfaces showed no significant differences in the time spent in each of the heart rate zones and higher concentrations of CK (+ 196.58; *p* > 0.05; CI 95% 66.54 to 326.61; ES: 1.645) were evidenced in the non-certified pitch surface. In response to a simulated match protocol, markers of post-exercise muscle damage may be reduced on accredited artificial turf fields. These insights can provide the opportunity to maximize the efficiency of training sessions and reduce the risk of injury during the season.

## Introduction

Football is experienced by actions of great intensity such as jumps, changes of direction, accelerations, decelerations and sprints along with moments of recovery^1,2^. Therefore, physical^3^ and physiological^4^ parameters influence the performance of football players. On the other hand, an influential external variable in the game is the sports surface. It has been proven that the state of the playing field affects the performance^5,6^ and the injury risk^7^ of the football player. The first comparative studies between surfaces were aimed at comparing the rate of injuries with respect to natural grass compared to artificial grass [(male match rate ratio 1.0 (95% CI 0.9–1.2); and female match rate ratio 1.2 (95% CI 0.8–1.8)]^8^.

Most of the research has focused on comparing the properties of artificial turf compared to natural grass, especially in terms of safety, with the risk of injury being the most analysed component^7–9^. Meanwhile, Andersson, Ekblom and Krustrup^10^ analysed the impact of these two surfaces on movement patterns in total distance (10.19 km, s = 0.19 vs. 10.33 km, s = 0.23), high-intensity running (1.86 km, s = 0.10 vs. 1.87 km, s = 0.14), number of sprints (21, s = 1 vs. 22, s = 2), both *p* < 0.05 on artificial turf than natural grass. Another line of research has been related to the lifecycle of the artificial turf, in order to control the variables that ensure the maximum durability of the surface. In this regard, the effect of the specification and maintenance^11^ related to the deterioration of the mechanical properties of the surface as a result of their exploitation has been more addressed^12^. The results of these studies have generated a great development in the last generation of artificial turf, allowing the existence of a large of combinations of structural components, resulting in different types of artificial turf fields. Although it may seem an unimportant fact, some authors believe that the differences between the different types may be greater than those between natural grass and artificial turf^13^. Previous investigations^14^ noted the significant differences between the different types of surface in their mechanical properties, focusing mainly on the support structure (sub-base and elastic layer). In this sense, it is very important to consider that the development and evolution of safe and quality artificial turf systems guarantee substantial savings in water consumption. Thereby, this situation consolidating the goal 6 in the 2030 Water and Agenda 'Ensure access to water and sanitation for all ', especially in underdeveloped and developing countries.

However, one of the areas that needs more study on the use of artificial turf in elite football is the influence on sport performance, which has been avoided by the scientific investigations so far. Some previous studies showed a clear influence of the playing surface (sand, asphalt and artificial turf) on physical parameters, physiological and psychological parameters. In this context, one physiological factor to consider is muscle damage. Different studies have evaluated muscle damage between very disparate surfaces, such as sand and grass or wood, showing that firm surfaces (e.g. grass or wood) generate greater muscle damage in eccentric movements, such as those used in football^15^. This muscle damage is less when faced with the same stimulus and the greater its recovery^16^.

On the other hand, this physiological variable has not been studied in artificial grass fields based on its mechanical properties. The mechanical properties may be in the correct parameters. An excess above or below the recommendations could negatively influence the interaction with the athlete. For example, a soft surface prevents the risk associated with repeated impacts, but on the contrary it would be associated with an excessive increase in physiological demand, earlier fatigue and risk of injuries associated with overload^17^. Consequently, the ideal would be to find the optimum point of the mechanical properties. For that reason, the aim of this study was to analyse the influence of the FIFA Quality PRO certification of artificial grass pitches on the physical and physiological performance and muscle damage in football players.

## Results

The two-way ANOVA test revealed differences between the quality of the surfaces and the half of the test in the variables evaluated (*p* < 0.05; Table 1). Results reveal a significant reduction in the sprint time of 12 m in the second half of the test compared to the first half in the FIFA Quality Pro certification field (− 0.08 s; CI 95% − 0.14 to 0.02; ES: 0.489). Similarly, the 12 m sprint times during the first half in the non-certified field were lower than the times obtained on the FIFA Quality Pro surface (− 0.10 s; CI 95% − 0.18 to 0.03; ES: 0.649). For the second half of the test, the average time of the 20 m sprints was longer on the FIFA Quality Pro surface (+ 0.13 s; CI 95% − 0.01 to 0.27; ES: 0.305). However, the average jump height was lower on the non-certified surface both in the first (− 2.00 cm; CI 95% − 3.76 to 0.23; ES: 0.297), as in the second half (− 2.01 cm; 95% CI − 4.17 to 0.16; ES: 0.274). Finally, the effort perceived by the players was higher in the first (+ 2.64; CI 95% 0.92 to 4.35; ES: 1.421) and the second half (+ 1.35; CI 95% − 0.02 to 2.72; ES: 0.637) of the test on the FIFA Quality Pro field. In this sense, the players showed a significant increase in the effort perceived in the second half of the non-certified field (+ 1.79; CI 95% 1.11 to 2.46; ES: 0.778).

**Table 1:** Mechanical properties of the selected artificial turf systems and reference values.

	StV (mm)	FR (%)	ER (%)
Reference Values*	4–10	60–70	n.a
FIFA Quality Pro Field	9.28 ± 0.10	63.02 ± 1.21	42.67 ± 1.70
Non-Certified Field	3.64 ± 0.41	38.62 ± 4.81	66.9 ± 2.96

*Reference values for FIFA Quality Pro field test requirements (FIFA, 2015). StV = Standard Vertical Deformation; FR = Force Reduction; ER = Energy Restitution; n.a. = not applicable.

**Figure 1:**
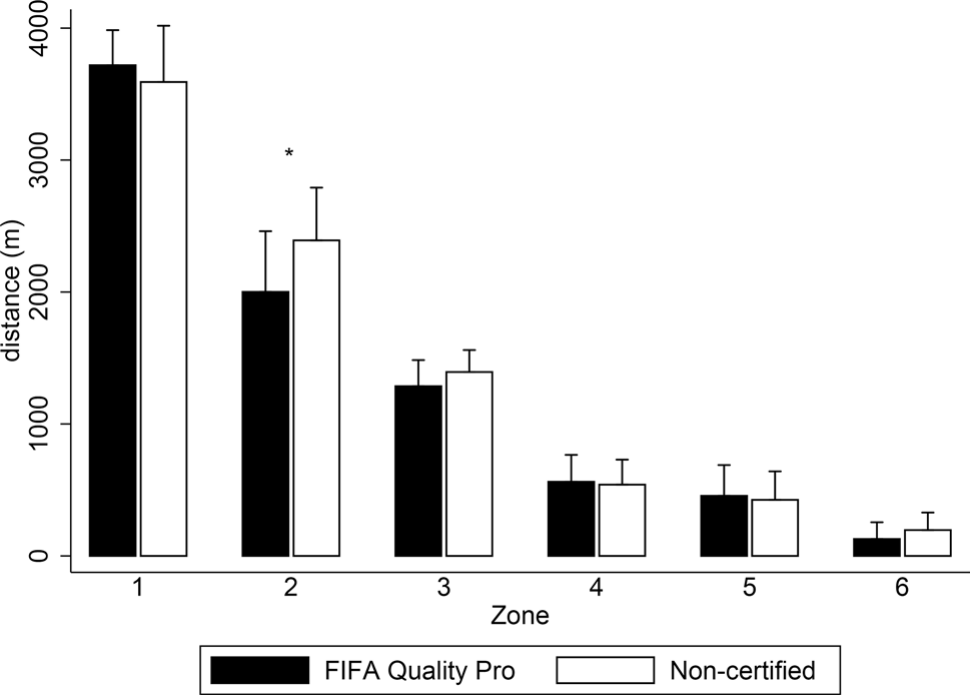
Activity profile during the BEAST_90_ in a FIFA Quality Pro and a non-certified artificial turf football field expressed as the total distance covered in different locomotor categories: Zone 1 (0–2 km h^−1^); Zone 2 (2–7 km h^−1^); Zone 3 (7–13 km h^−1^); Zone 4 (13–18 km h^−1^); Zone 5 (18–21 km h^−1^); and Zone 6 (> 21 km h^−1^). *Significant differences between FIFA Quality Pro and non-certified fields (*p* < 0.05).

**Figure 2:**
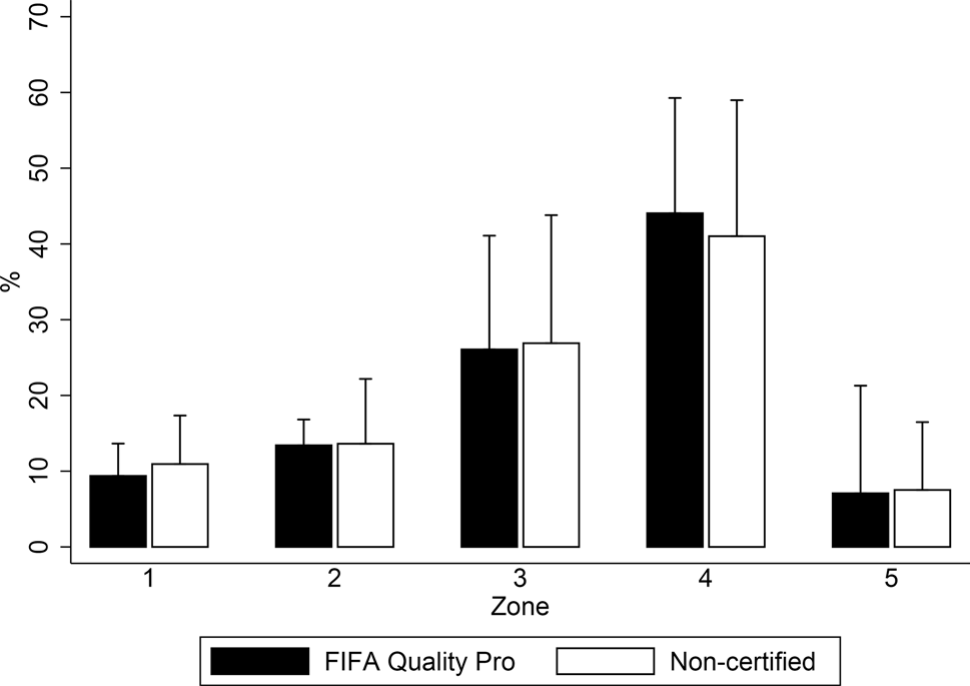
Distribution of heart rate expressed as the percentage of game time taken in the different ranges of HR_MAX_ during a simulated game test (BEAST_90_) in a FIFA Quality Pro (Black) and a non-certified (White) artificial turf football field.

The results of external load showed a greater distance travelled over the non-certified field (+ 386.01 m; CI 95% 34.93 to 737.09; ES: 0.710; Table 2). Nevertheless, the number of sprints was lower compared to the FIFA Quality Pro surface (− 8.63; CI 95% − 17.03 to − 0.22; ES: 0.843). No significant differences were found between surfaces in high-intensity distances (*p* > 0.05; Fig. 1), except for distance in zone 2 (− 390.39 m; CI 95% − 467.06 to − 313.71; ES: 0.91). In relation to internal load, players showed higher HR_MEAN_ on the non-certified field in absolute (+ 3.60 b.p.m.; CI 95% 0.31 to 6.89; ES: 0.470) and relative terms (+ 1.90%; CI 95% 0.13 to 3.67; ES: 0.490). The comparative analysis between surfaces did not show significant differences in the time spent in each of the heart rate zones (*p* > 0.05; Fig. 2).

**Table 2:** Physical demands and RPE scale during the first and second half of BEAST_90_ in a FIFA Quality Pro and non-certified artificial turf football fields.

		FIFA Quality Pro		Non-Certified	
		First Half	Second Half	First Half	Second Half
Mean	12 m sprint (s)	2.10 ± 0.15*#	2.02 ± 0.18	2.00 ± 0.16	2.01 ± 0.18
	20 m sprint (s)	3.30 ± 0.34	3.19 ± 0.40#	3.11 ± 0.26	3.06 ± 0.28
	CMJ (cm)	38.12 ± 6.86#	37.80 ± 7.62#	36.12 ± 6.58	35.79 ± 7.01
	Goals (n)	3.80 ± 0.53	3.79 ± 0.72	3.72 ± 0.72	4.05 ± 0.54
	RPE (u.a.)	14.20 ± 1.57#	14.70 ± 1.79#	11.56 ± 2.14*	13.35 ± 2.45
Δ% first and last lap	12 m sprint (s)	0.09 ± 0.27	0.17 ± 0.14	0.06 ± 0.11*	0.14 ± 0.08
	20 m sprint (s)	0.10 ± 0.26	0.10 ± 0.22	− 0.01 ± 0.13	0.03 ± 0.08
	CMJ (cm)	1.25 ± 3.83	1.21 ± 4.88	1.58 ± 2.82	0.24 ± 2.02
	Goals (n)	− 0.92 ± 1.75	− 0.38 ± 1.80	− 1.00 ± 3.16	− 0.18 ± 1.15
	RPE (u.a.)	− 6.62 ± 3.04	− 5.69 ± 2.69	− 5.00 ± 5.585.58	− 5.75 ± 1.84

*Differences between 1st half and 2nd half; # Differences between FIFA Quality Pro and non-certified.

The assessments made before, during and after the BEAST_90_ showed a greater perceived effort (+ 2.32; CI 95% 0.27 to 4.39; ES: 7.093) and a lower Flicker score (− 1.49; CI 95% − 2.79 to − 0.19; ES: 0.113) at half-time in the FIFA Quality Pro field compared to the non-certified surface (*p* < 0.05; Table 3). Once the test was finished, players revealed lower levels of force in the upper train (+ 4.11; CI 95% 0.75 to 7.48; ES: 0.572), as well as higher concentrations of CK (+ 196.58; CI 95% 66.54 to 326.61; ES: 1.645) in the field with the non-certified surface (*p* < 0.05). In this field, BEAST90 test caused a significant increase in blood lactate concentration (+ 3.94; CI95% 1.15 to 6.72; ES: 1.496), as well as a reduction of the PEF (− 1.39; CI95% − 2.11 to − 0.89; ES: 0.742) compared to the baseline measurements (*p* < 0.05). CK (+ 105.58; CI 95% − 4.12 to 215.28; ES: 1.168) and RPE values (+ 9.77; CI 95% 8.07 to 11.62; ES: 7.093) increased in the FIFA Quality Pro field (Table 4).

**Table 3:** External and internal load of BEAST_90_ in a FIFA Quality Pro and non-certified artificial turf football fields.

	FIFA Quality Pro	Non-Certified
External Load		
Total Distance (m)	8151.32 ± 638.93	8537.33 ± 448.47*
Maximum Speed (kmh^−1^)	26.83 ± 2.81	27.25 ± 2.59
Number of sprints (n)	20.13 ± 10.67	11.5 ± 9.78*
Accelerations Zone 1 (n)	140 ± 28.17	162.13 ± 22.44*
Accelerations Zone 2 (n)	42.13 ± 22.92	28.88 ± 10.68
Accelerations Zone 3 (n)	1.13 ± 1.36	0.5 ± 0.76
Decelerations Zone 1 (n)	144 ± 33.65	147.13 ± 36.76
Decelerations Zone 2 (n)	17.63 ± 15.36	13 ± 8.12
Decelerations Zone 3 (n)	0.63 ± 1.77	0 ± 0
Internal Load		
HR_MEAN_ (b.p.m.)	148.4 ± 8.53	152 ± 6.78*
HR_MAX_ (b.p.m.)	191.3 ± 10.83	185.9 ± 16.35
HR_MEAN_ (%)	74.4 ± 4.35	76.3 ± 3.40*
HR_MAX_ (%)	95.8 ± 5.43	93.2 ± 8.07

HR: Heart Rate *Significant differences between FIFA Quality Pro and non-certified fields (*p* < 0.05).

## Discussion

This research exposes the influence of the mechanical property certification of the artificial turf football fields on the psychophysiological sport performance, which is evaluated through the BEAST_90_ test. The evaluation of sport performance based on field certification (comparing a FIFA Quality Pro and a non-certified surface) makes this study a pioneer in the scientific area. The main finding of this research was that a different mechanical behaviour between artificial turf pitches produced different psychophysiological responses in football players in a simulated game protocol (BEAST_90_ test).

Previous studies have already demonstrated the influence of the physical and physiological response between natural and artificial turf football fields^5,18^, or among different artificial turf surfaces^19^. However, the psychophysiological response based on the certification of the pitch had not been evidenced previously. Burillo et al.^20^ consider it necessary to raise awareness and demonstrate quality standards to achieve sustainability (economic, physical performance and health of the athlete) in the football world. The FIFA Quality Pro surface had the highest levels of StV and FR, complying with the most demanding certification values. This aspect guarantees a less hard playing field with optimal mechanical properties for the practice of elite football^19^. The mechanical differences between surfaces ranged between 54.94% and 63.17%, coinciding with results of previous studies that affirm that the differences between artificial turf fields with different structures can be greater than the variation between an artificial turf field and a natural one^21^.

The inclusion of the ER values in this study provides more details about hardness and the absorption capacity of the surface^19^. A reduced cushioning capacity and an increase in the energy of restitution of the artificial turf surface can modify the performance of football players^19^, especially in high-intensity actions as the results of this study have evidenced on the external load. ER measures the energy returned to the player from the surface after an impact^22^. This mechanical property of the surface has been shown to be decisive in the physical performance of the players, improving the sprint times^19^. In this case, the uncertified field revealed a 56.78% increase in energy returned from the playing surface after an impact. This result may explain the best times in 12 m and 20 m sprints in the non-certified football field. This suggests that the percentages of FR and StV were high enough (according to current regulation) to generate an increase in the sprint times derived from the reduction of the reaction forces as a result of the partial absorption of the energy applied in FIFA Quality Pro artificial turf field^23^. Certainly, the lower contact time during running on the non-certified artificial turf system^21^ and the less frequent reuse of stored elastic energy^16^ of the artificial turf systems with a higher damping capacity can explain the differences in the covered distance and sprint times found in the present research. However, a higher jump height was identified on the FIFA Quality Pro artificial turf field, probably because of the potentiation effect to compensate the degradation of elastic energy and lower muscle damage caused by a higher force reduction on the surface^15^.

This reduction in the energy returned by the surface could explain the higher RPE values found during the test in the FIFA Quality Pro in comparison to the non-certified field. This was already manifested by previous authors who demonstrated that the perceived effort is higher in comfortable and softer playgrounds, although the evidenced physiological response is usually similar^19,24^. A lower muscle-sinew efficiency^25^ or a higher hip and knee flexion^26^ could clarify this result due to a higher energy outlay on surfaces with a higher impact reduction. However, the physiological load was slightly higher in the non-certified field (76.30 vs 74.40% HR_MEAN_). This result shows that the perceived demand is more related to the difficulty in running (external load) than to the physiological responses (internal load)^24^, since no differences were found in the blood lactate concentrations and the time spent in the different heart rate zones. The greater perceived effort on the surface with greater damping capacity was more evident at the end of the first part of the test. A lower running efficiency^27^ or a worse use of the generated energy^28,29^ could determine an early perceived fatigue in the FIFA Quality Pro field. It is possible that a higher degree of muscular engagement and activation on the softest surface may have increased the football players’ lower-limb muscle awareness and perception of fatigue^30^.

Similar high-intensity actions (speeds over 21 km h^−1^; accelerations and decelerations) were found on both surfaces. However, higher total distances travelled in the non-certified field were found. In addition, the HR_MEAN_ was significantly higher in this field both in absolute (b.p.m.) and relative (%) values. In the same vein, Sánchez-Sánchez et al.^31^ showed that the hardest artificial turf surfaces (with less normative adjustment) produce certain improvements in some performance parameters, such as total distance travelled, and as in our study, they found no differences in heart rate, blood lactate or high-intensity accelerations among surfaces with better or worst adjustment to FIFA regulations. As stated by Nédélec et al.^18^ the hardest playing surfaces do not produce any extra load of muscular work. Thereby, these actions with greater physical demands could obtain the same values regardless of the type or the normative adjustment of the surface. However, the non-certified field revealed a higher heterogeneity between the first and the second half of the test in the physical performance, probably due to a higher muscle damage on this surface^15,16^.

Regarding the cortical response, we found how in the middle test the rate of perceived exertion in the FIFA Quality Pro was higher than on the non-certified field, nevertheless the cortical arousal was not in line with perceived exertion, since on the FIFA Quality Pro field the cortical arousal remained close to the basal values and in the non-certified presented a decreased value. This result showed the higher negative impact of the non-certified field at cortical level^32^, but after the test, the cortical response was similar in both fields, showing the necessity for future research to explain this effect. Linked to the cortical, muscular control and strength manifestation we found how the FIFA Quality Pro field allows players to maintain strength of muscle not directly implied in the activity (HIS) and respiratory muscles (spirometry variables), and a tendency to increase strength manifestation of muscle directly implied in the activity (horizontal jump test), while this tendency was not shown in the non-certification field. The maintained or even increased strength manifestation could be explained by the increased sympathetic modulation due to the eliciting characteristic of the BEAST_90_ test^33^, the lower blood lactate concentration, as well as the lower muscular destruction (CK) evaluated in the FIFA Quality Pro field^34^. In this context, the lower CK values reported in the FIFA Quality Pro field highlighted the lower muscular impact of this field in the players’ muscle system, a fundamental fact to allow them a shorter recovery period after training and competitions as well as a decreased injury risk^35^. According to previous studies, softer and accredited artificial turf fields can reduce the impact received during the test and protect against injuries associated with the impacts compared to harder artificial turf surfaces^17^.

This study has several limitations. Firstly, many of the significant differences found based on the p values (*p* < 0.05) include values above and below 0 in the CI 95%. In these cases, the upper or lower value is very close to 0, however, the results may be considered with caution and may be contrasted with larger samples in future studies. Secondly, the data have been obtained with amateur football players, therefore, they cannot be replicated in professional football.

## Conclusions

In conclusion, FIFA Quality Pro fields, in comparison with fields that do not comply with the surface performance parameters, could reduce the impact on the muscle, musculoskeletal strain and therefore, improve the training stimulus. A higher force reduction and lower energy restitution diminished the muscle damage of the players, which could accelerate the recovery process after the training sessions and matches, and protect more protection against injuries compared to non-certified harder artificial turf surfaces, especially at a time in the season where a reduced musculoskeletal loading would be necessary (i.e. congested competition schedule). These insights can provide the opportunity to maximize the efficiency of the training sessions and reduce the risk of injury during the season. A strategy to be followed by coaches and players who practice football on non-certified artificial turf pitches could be to perform the plyometric and eccentric exercises characteristic of soccer on sand, since this surface has lower restitution energy and greater force reduction^6^.

## Material and methods

### Subjects

The study comprised a convenience sample of 15 healthy male players from a university football team, all of whom volunteered to take part in this study. Players trained as a team 3 days per week for approximately 2 h per training session and competed in one weekly match in the Spanish University League according to previous studies^36^. Players were recruited via the football coach, who explained the study to the whole team and that participation was voluntary. The mean (± SD) age, height, body mass, and competitive playing age were 21.2 ± 1.4 years; 1.78 ± 0.43 m; 79.1 ± 8.3 kg; and 10.6 ± 3.5 years, respectively. Players completed a YY intermittent recovery test (Level 1) to establish their maximum heart rate (HRmax) through a heart rate monitor (Polar Team System, Kempele, Finland) attached to their chest. Each participant provided written informed consent before any testing began, based on the last version of the Helsinki Declaration and was approved by the Clinical Research Ethical Committee of European University of Madrid, Spain (CIPI36/2019).

### Design

#### Mechanical properties of the surfaces

**Figure 3:**
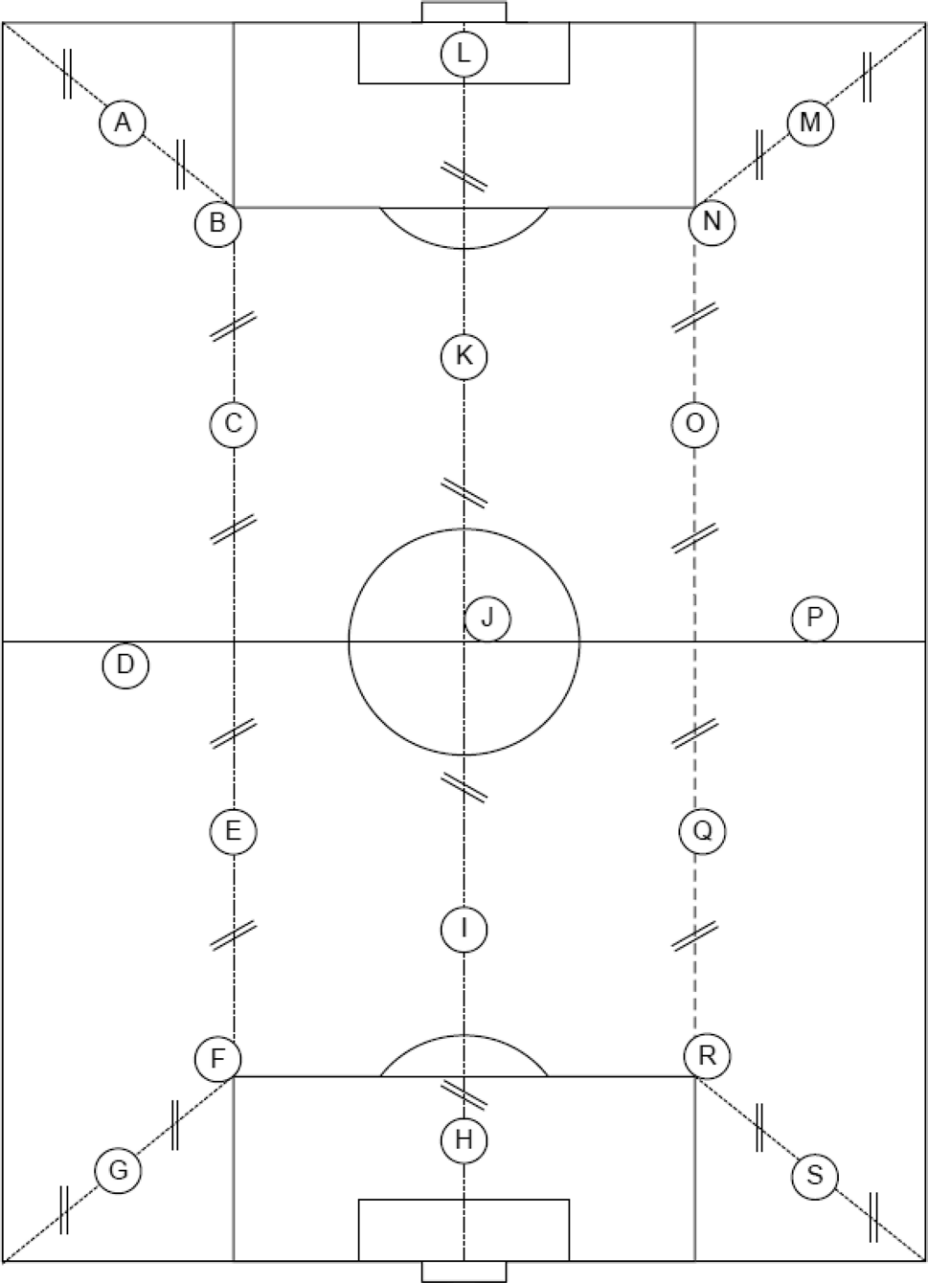
Test zones according to regulation FIFA Test Methods 2015 (FIFA, 2015).

The mechanical properties related to the surface–player interaction were assessed in the two surfaces selected for this study (one without any type of certification and one with FIFA Quality Pro certification). Regardless of the weather situation and age, a field may not meet FIFA Quality Pro certification requirements for several reasons, including product quality or failure to install performance fillers, lack of maintenance or usage higher than recommended. In either case, the result on the characteristics of the surfaces is the same, a surface with poor quality or worn materials and mechanical properties outside of the recommendations for a safe and functional game. The selection criteria were that both surfaces had a similar age and weather, one of them meeting FIFA requirements and the other not. In situ tests according to FIFA Test Methods (FIFA, 2015) and previous studies^14,19^ were performed. The analysed variables were force reduction (FR-%) according to FIFA Test Methods 04a, standard vertical deformation (StV-mm) according to FIFA Test Methods 05a and energy restitution (ER-%) according FIFA Test Methods 13 (Table 4). All variables are related with the surfaces’ response to an impact, and they were evaluated using an Advanced Artificial Athlete (Wireless Value, Emmen, The Netherlands). A mass with an incorporated spring, weighing in total 20 kg, was dropped on the surfaces. The acceleration of the mass from the output until after surface impact was transmitted through a specific software to a mobile phone (BlueImpact R9G, Wireless Value, Emmen, The Netherlands) to extrapolate the collected data to the variables evaluated. This procedure was repeated two more times at intervals of 60 ± 10 s, resulting in a total of three impacts. For the statistical analysis, the mean value between the second and the third impact was registered. In turn, this was done inside the 19 zones specified by FIFA Test Methods 2015 regulations (Fig. 3).

**Table 4:** Effect of BEAST_90_ on attention RPE, physical, respiratory and biomarker parameters in a FIFA Quality Pro and non-certified artificial turf football fields.

		Pre (1)	Medium (2)	Post (3)	Post 24 h (4)
FIFA Quality Pro	CFFT (Hz)	35.14 ± 3.19^2^	35.87 ± 3.87*	35.54 ± 3.86	
	RPE (u.a.)	7.00 ± 0.71^2,3^	16.85 ± 2.08*	16.77 ± 2.05	
	Hand force (N)	41.02 ± 7.40	41.46 ± 8.78	41.27 ± 8.69*	
	Horizontal Jump (cm)	178.23 ± 22.91^3,*^	178.62 ± 23.42	184.00 ± 19.98	
	FVC	5.02 ± 1.13		4.81 ± 1.49	
	FEV1	3.96 ± 0.91		3.57 ± 1.17	
	PEF	10.65 ± 3.65		9.21 ± 3.70	
	Blood Lactate (mmol L^−1^)	2.81 ± 1.17*		4.88 ± 2.87	
	CK (IU− L^−1^)	78.73 ± 49.05^4^		184.31 ± 131.78^4,*^	525.08 ± 469.90
Non-Certified	CFFT (Hz)	36.20 ± 3.06	37.36 ± 3.94	36.75 ± 2.75	
	RPE (u.a.)	6.77 ± 0.83^2,3^	14.52 ± 2.78^3^	16.69 ± 2.14	
	Hand force (N)	41.78 ± 7.12^3^	41.71 ± 8.03	37.67 ± 7.28	
	Horizontal Jump (cm)	194.66 ± 16.89	210.59 ± 70.39	187.57 ± 12.51	
	FVC	4.96 ± 0.72		5.09 ± 0.71	
	FEV1	4.19 ± 0.58		3.66 ± 1.02	
	PEF	10.27 ± 2.25^3^		8.88 ± 1.52	
	Blood Lactate (mmol L^−1^)	1.73 ± 0.59^3^		5.67 ± 4.66	
	CK (IU L^−1^)	86.71 ± 58.10^3^^,^^4^		283.29 ± 180.85^4^	681.80 ± 419.25

*Significant differences between FIFA Quality Pro and non-certified artificial turf fields (*p* < 0.05). 1, 2, 3, 4 Significant differences at half-time (2), immediately post-test (3), 24 h post-test (4) (*p* < 0.05). CFFT: critical flicker fusion threshold; RPE: rated of perceived exertion; FVC: forced vital capacity; FEV1: volume exhale at the end of the first second of forced expiration; PEF peak expiratory flow; CK creatinkinase.

The geographical proximity (centre region of Spain) of the two artificial turf football fields guaranteed similar climatic conditions. Tests were carried out under 22–24.5 °C temperature.

#### BEAST90 protocol

Players were required to attend four testing sessions: two familiarity sessions and two actual full tests, one on every surface. During the familiarity session all players went with a person that would be with them during the test in order to register their times and scores in both the jump and shot on goal, in which they walked and got to know the different actions that they would need to do during the circuit. The familiarity session was performed on the same football pitch where one of the trial runs was done according to previous studies^37^. All the people involved in the familiarization sessions participated as staff members in charge during the measurement days. All of them were graduates in sports sciences, masters in sports performance or Ph.D. in Sports Sciences, with previous experience in the management and use of the instruments and knowledgeable about the protocol.

The day before the experimental test, it was recommended to the players to not carry out any kind of exhausting activity, as well as to maintain their usual eating habits; they were also advised to use the same footwear in the two systems being assessed^37^. All subjects were tested in both conditions by randomized cross-over study. Players arrived at the football field at 09∶00 a.m. A Global Positioning System (GPS, WIMU Pro, RealTrack System, Almería, Spain) and heart rate band with values averaged every 5 s (Garmin, Switzerland Gmbh) were incorporated on each football player’s back and trunk respectively. Before the beginning of the different tests, participants carried out a standard warm-up, which included exercises such as 5 min of continuous running, 5 min of exercises of articulation mobility and three sprints of 30 m, increasing the intensity, with a recovery process of 2 min. Stretching exercises were not carried out during the warm-up. At 10∶00 a.m. players started the performance test and were verbally instructed to apply the maximum effort during the tests^37^.

**Figure 4:**
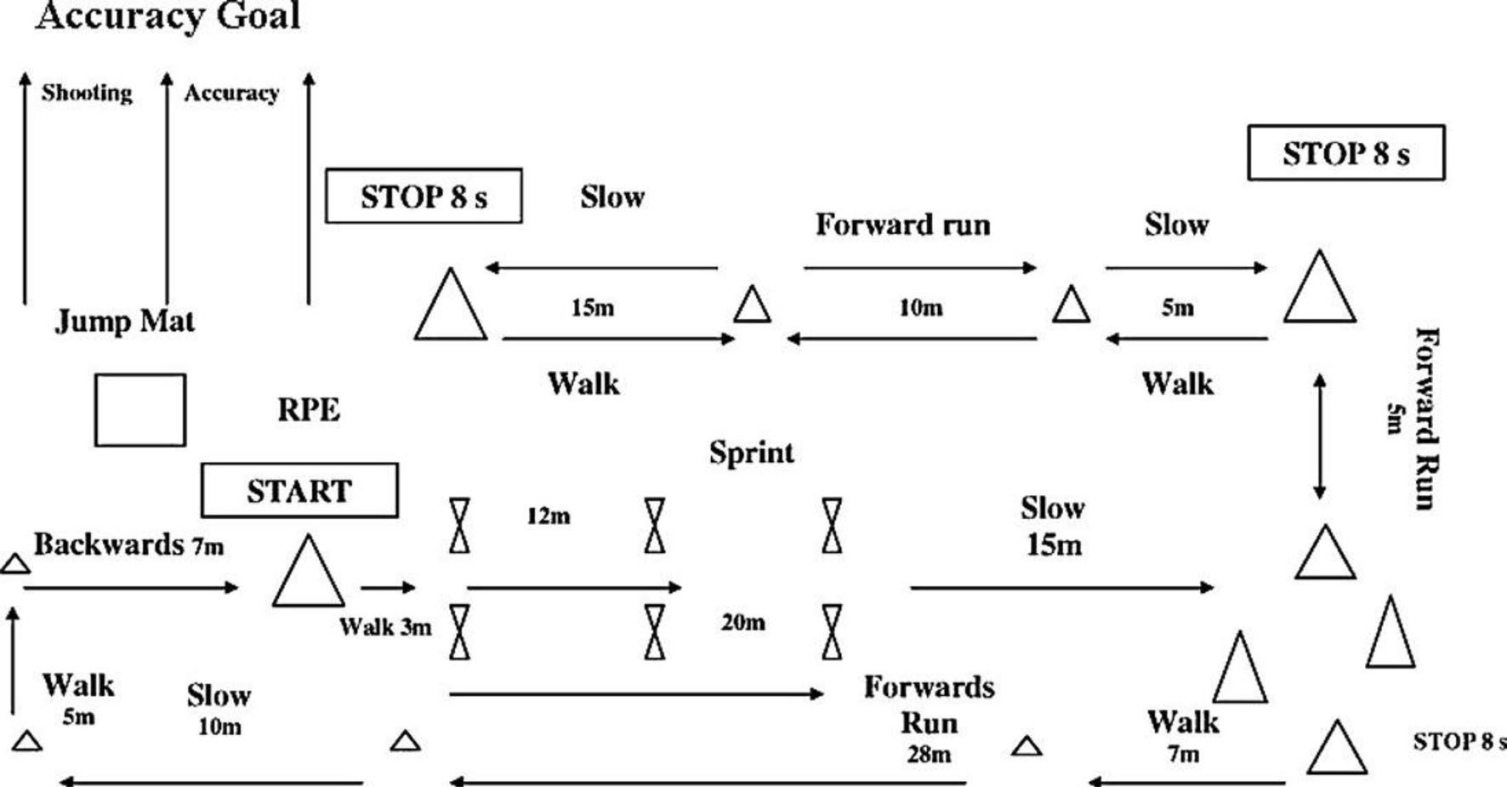
Schematic BEAST_90_ Protocol^37^.

The simulated football protocol chosen to induce fatigue was the Ball-sport Endurance and Sprint Test^37^ (BEAST_90_) (Fig. 4). The chosen test is the test that best reproduces the physical demands produced during a football match^38^. The test consists of repeated circuits during two 45 min halves, separated by 15 min of rest, including sprinting (12 m and 20 m), running at approximately 75% of maximum effort, jogging/decelerating, walking, backward jogging, slaloming between cones and kicking a football^37^. Between circuits, participants performed three maximal countermovement jumps (CMJs). For the registration of the flight time during the jump and determining CMJ performance an OptoJump–Microgate optical measurement system (Optojump, Bolzano, Italy) was used on a concrete surface. Likewise, for the control of the sprints, a set of two timing gates RaceTime (Microgate, Bolzano, Italy) were used. For the shots on goal control, a spreadsheet was used by each of the players’ assistants. To quantify the total time that elapsed during the test and pauses, every player assistant used a chronometer Seiko 5141 (Seiko Watch Corporation, Tokio, Japan). Total distance (m), distance Zone 1 (m; 0–2 km·h^–1^), distance Zone 2 (m; 2–7 km·h^−1^), distance Zone 3 (m; 7–13 km h^−1^), distance Zone 4 (m; 13–18 km h^−1^), distance Zone 5 (m; 18–21 km h^–1^), distance Zone 6 (m; > 21 km h^–1^), number of sprint (n), accelerations Zone 1 (n; 0–2 m s^2^), accelerations Zone 2 (n; 2–3 m s^2^), accelerations Zone 3 (n; > 3 m s^2^), decelerations Zone 1 (n; 0–2 m s^2^), decelerations Zone 2 (n; 2–3 m s^2^) and decelerations Zone 3 (n; > 3 m s^2^) were registered. On the other hand, Heart Rate Mean (HR_MEAN_; b.p.m.), Heart Rate Max (HR_MAX_; b.p.m.), Heart Rate Mean (HR_MEAN_; %), Heart Rate Max (HR_MAX_; %) and the time spent in the different heart rate zones [Heart Rate Zone 1 (min; 50–59% HR_MAX_); Heart Rate Zone 2 (min; 60–69% HR_MAX_); Heart Rate Zone 3 (70–79% HR_MAX_); Heart Rate Zone 4 (80–89% HR_MAX_); Heart Rate Zone 5 (90–100% HR_MAX_)] were monitored. The protocol was developed individually by each participant (one by one).

#### Physiological tests

Baseline and immediately oost-intervention assessment of the following tests was carried out in every football field analysis: physiological protocol rating of perceived exertion (RPE), Borg 6–20 scale; blood lactate concentration, taking a sample of 5 μl capillary blood from a subject’s finger and analysed with the Lactate Pro II Arkay, Inc. system (Kyoto, Japan); blood creatinkinase concentrations, taking a sample of 32 µl capillary blood from a finger and analysed using the Reflotron Plus system, (Roche Diagnostics S.L. Sant Cugat del Vallès, Barcelona); cortical arousal and fatigue of the central nervous system (CNS) was measured by critical flicker fusion threshold (CFFT 12,021; Lafayette Instrument Company, Lafayette, IN) by the average of 5 incremental tests (20 to 100 Hz), according to previous research^39^; lower body muscular strength manifestation by employing a horizontal jump test; subjects performed two maximal horizontal jumps as a previous report informed, and the best attempt was used for the statistical analysis^32^; isometric hand strength (IHS) by a grip dynamometer (Takei Kiki Koyo, Tokyo, Japan); and spirometry variables of forced vital capacity (FVC), volume exhale at the end of the first second of forced expiration (FEV1) and the peak expiratory flow (PEF) using a QM-SP100 (Quirumed, Spain) spirometer in a maximum inhale–exhale cycle, according to previous research^40^.

### Statistical analysis

Results are presented as means ± standard deviations. Normality and homogeneity of the variance were assumed after the Kolmogorov–Smirnov test and the Levene’s statistical analysis. Three comparison analyses between performance variables were developed through repeated measures ANOVA. For physical demands and Borg scale during the first and second half of BEAST90 two-way repeated measures ANOVA were performed (first and second half vs FIFA Quality Pro-field and no-certification field). The variables related with external and internal load of BEAST90 were compared by one-way repeated measures ANOVA (FIFA Quality Pro-field and no-certification field). Finally, attention, RPE, physical, respiratory and biomarkers parameters were compared by one-way repeated measures ANOVA in each field. The observations in this analysis were baseline, measures between each part of BEAST_90_, post BEAST_90_ and 24 h post. In all cases, Bonferroni post hoc was used. Confidence interval (CI of 95%) and effect sizes (Cohen d, ES) were calculated and defined as follows: trivial, < 0.19; small, 0.2–0.49; medium, 0.5–0.79; large, > 0.8^41^. Data were analysed with the statistical software SPSS v21.0 (IBM, Chicago, IL). The level of significance was established at *p* < 0.05.
